# Type 1 Diabetes and Addison’s Disease: When the Diagnosis Is Suggested by the Continuous Glucose Monitoring System

**DOI:** 10.3390/children8080702

**Published:** 2021-08-14

**Authors:** Francesco Vinci, Giuseppe d’Annunzio, Flavia Napoli, Marta Bassi, Carolina Montobbio, Giulia Ferrando, Nicola Minuto

**Affiliations:** 1 Department of Neuroscience, Rehabilitation, Ophtalmology, Genetics, Maternal and Child Health, University of Genoa, 16132 Genova, Italy; francesco.vinci201@gmail.com (F.V.); martabassi.ge@gmail.com (M.B.); carolina.montobbio@gmail.com (C.M.); tulia.ferrando@gmail.com (G.F.); 2 Department of Pediatrics, IRCCS Istituto Giannina Gaslini, University of Genoa, 16147 Genova, Italy; 3 Department of Pediatric Clinic and Endocrinology, Regional Center for Pediatric Diabetes, IRCCS Giannina Gaslini, 16147 Genoa, Italy; giuseppedannunzio@gaslini.org (G.d.); flavianapoli@gaslini.org (F.N.)

**Keywords:** adrenal insufficiency, type 1 diabetes, continuous glucose monitoring, hypoglycemia, Schmidt’s syndrome

## Abstract

Our objective is to emphasize the important role of continuous glucose monitoring (CGM) in suggesting adrenal insufficiency in patients affected by type 1 diabetes. We describe an adolescent girl with type 1 diabetes and subsequent latent Addison’s disease diagnosed based on a recurrent hypoglycemic trend detected by CGM. In patients with type 1 diabetes, persistent unexplained hypoglycemic episodes at dawn together with reduced insulin requirement arouse souspicionof adrenal insufficiency. Adrenal insufficiency secondary to autoimmune Addison’s disease, even if rarely encountered among young patients, may be initially symptomless and characterized by slow progression up to acute adrenal crisis, which represents a potentially life-threatening condition. Besides glycometabolic assessment and adequate insulin dosage adjustment, type 1 diabetes needs prompt recognition of potentially associated autoimmune conditions. Among these, Addison’s disease can be suspected, although latent or paucisymptomatic, through periodic and careful evaluation of CGM data.

## 1. Introduction

Continuous glucose monitoring (CGM) is today an essential tool for a better glycometabolic control in type 1 diabetes. It is increasingly used, especially among young patients, and is gradually replacing traditional self-monitoring of blood glucose. It is a safe and effective method that is becoming essential to the achievement, improvement, and maintenance of optimal glycemic levels. CGM allows for a better characterization and prompt recognition of glycemic variability by means of trend arrows, which indicate the predicted variation of glycemic values over time, including asymptomatic hypoglycemic events [1,2,3,4].

On the other hand, associated autoimmune diseases, other than celiac disease and autoimmune thyroiditis, albeit infrequently encountered, still represent a risk factor for correct diabetes management, particularly in case of delayed detection or misdiagnosis [5]. Among these autoimmune disorders, antibody-mediated adrenal insufficiency (or Addison’s disease) is a relatively rare (1% or less) comorbidity of type 1 diabetes, which may be initially symptomless and characterized by slow progression up to acute adrenal crisis (Addisonian crisis), a potentially life-threatening condition [6,7,8,9,10,11,12,13,14,15,16,17,18,19]. In the past few years, CGM was used to monitor the glucose trend in those patients already diagnosed with adrenal insufficiency [7], but not usually as an early and handy red flag for this specific condition.

Autoimmune polyendocrine syndrome type 2 (APS 2), or Schmidt’s syndrome [16], is a complex polygenic disorder associated with the presence of specific class II HLA, and Addison’s disease is the predominant and most stable component (80–90% of cases). We describe the case of a young girl with long-standing type 1 diabetes and Hashimoto’s thyroiditis who developed an antibody-mediated paucisymptomatic adrenal insufficiency clinically suspected because of a recurrent hypoglycemic trend detected through analysis of CGM data.

## 2. Case Report

In April 2019, a 13-year-old girl with type 1 diabetes since the age of 10 was first admitted to our regional center for glycometabolic assessment. Medical history was positive for type 1 diabetes diagnosed without ketoacidosis and treated in another children’s hospital; insulin requirement was 0.45 U/kg/day by multiple subcutaneous injections, and glucose monitoring was performed by CGM Dexcom^®^ G5 (Dexcom, Inc., San Diego, CA, USA). One year before the first clinical evaluation at our center, insulin requirement was 0.8 U/kg/day. Family history was positive for autoimmune diseases such Hashimoto’s thyroiditis, vitiligo, and psoriasis. Physical examination displayed a Tanner stage 3: breast development was stage 3 and pubic hair development was stage 2. Height was 156.1 cm (33rd percentile) and weight was 39.4 kg (14th percentile). Medical evaluation was normal except for a slightly dark skin tone (similar to her father), shuffling gait, and rounded shoulders. The parents reported a significant, otherwise unjustified decline in school performance over the last few months. Laboratory evaluation showed good glycometabolic control (HbA1c levels, 7.1%, 54 mmol/mol) and primary hypothyroidism due to autoimmune thyroiditis (FT4, 10.8 pmol/L (normal range, 14.3–26.2); thyroid-stimulating hormone, 7.16 mU/L (normal range, 0.2–4.2 mU/L); antithyroperoxidase antibodies, 128 UI/mL (normal range, 0–34 UI/mL); antithyroglobulin antibodies, <10 UI/mL (normal range, 0–115 UI/mL) [18]. L-thyroxine therapy was started; no changes in the clinical picture appeared after restoration of normal thyroid function.

CGM data showed 52% of time spent in the euglycemic range in the last 14 days (desired value,>70% or >16 h, 48 min per day), 2% of time spent in hypoglycemia (desired value, <4% or <1 h per day), 46% of time spent in hyperglycemia (desired value, <25% or <6 h per day) [20]. The nocturnal glucose trend showed a frankly hyperglycemic pattern from midnight to 4:00 a.m., followed by a gradual decrease in glucose trend with recurrent hypoglycemia near dawn. The patient’s parents reported that this tendency often required the administration of either simple or complex carbohydrates right before bedtime in order to avoid glycemic drops in the first morning hours.

**Figure 1:**
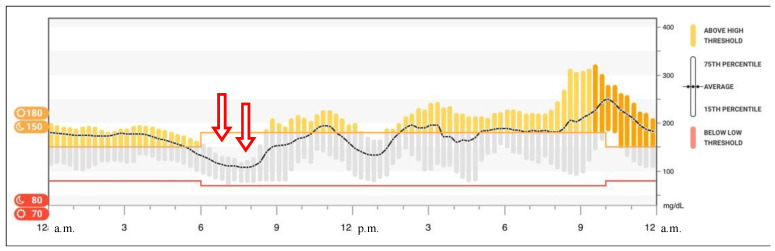
Glucose data reproduced from Dexcom^®^ CGM Clarity software (Dexcom, Inc., San Diego, CA, USA), the graph shows the glucose trend averaged over 14 days. Arrows indicate the sudden switch from hyperglycemia to hypoglycemia near dawn.

Based on these findings, basal insulin dosage was reduced (from 10 to 8 IU of degludec insulin per day) with a subsequent significant increase in time spent in hyperglycemia and without a decrease in time spent in hypoglycemia (Figure 1). One year before, CMG data had not shown any recurrence of hypoglycemia near dawn.

Three months later, the specific tendency toward high glucose levels in the first hours of the night and the subsequent sudden switch to hypoglycemia near dawn was confirmed despite basal insulin dosage reduction. Total insulin daily need was slightly reduced to 0.40 U/Kg per day, while weight was unchanged. CGM data showed unchanged time spent in the euglycemic range in the last 14 days and a slight increase in time spent in hypoglycemia (3%, desired value, <4% or <1 h per day).

The girl complained of moderate fatigue and daily asthenia needing afternoon resting. Due to the clinical symptoms, tendency toward hypoglycemic events at dawn, and low insulin requirement in long-standing type 1 diabetes, celiac disease, as the most common cause of unexplained hypoglycemic events, was ruled out. While clinical evaluation revealed a normal hydration state and skin hyperpigmentation, adrenal axis evaluation showed baseline morning (at 8.00) ACTH levels and cortisol levels of 377.3 pmol/L (normal range, 1.6–13.9) and 19.32 nmol/L (normal range, 68.2–538.2), respectively, which confirmed the diagnosis of primary adrenal insufficiency. Laboratory tests showed normal values of serum electrolytes, iron balance, liver and lipid profile, and absence of inflammatory markers. As a demonstration of the total absence of endogenous insulin production, the C-peptide was found to be <0.01 (normal range,1.1–4.4 ng/mL). Oral hydrocortisone at 23 mg/m^2^/day divided in 3 daily doses was started, followed by mineralocorticoid therapy with fludrocortisone (0.1 mg/day) because of high plasma renin concentration (1.24 pmol/L, normal range, 0.05–1.05) [8].

Abdominal magnetic resonance imaging showed normal anatomy of adrenal glands. Intradermal injection with the Mantoux procedure was negative, and serum levels of very-long-chain fatty acids were normal. The antiadrenal cortex antibodies were positive, and Addison’s disease was confirmed. The patient was discharged with oral hydrocortisone, 14 mg/m^2^/day, and fludrocortisone, 0.05 mg/day, with insulin requirement progressively increased up to 1 U/Kg/day. The clinical conditions improved with the disappearance of asthenia and hypoglycemia, followed by an improvement of the glycometabolic profile.

**Figure 2:**
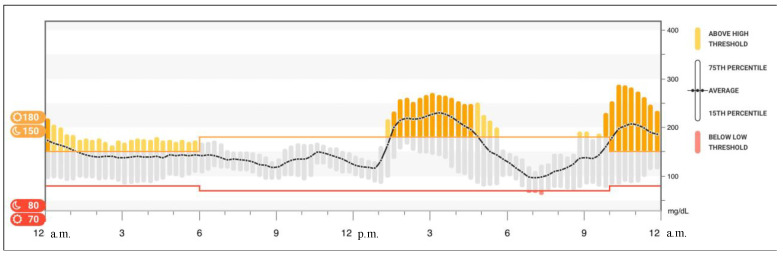
Glucose data reproduced from Dexcom^®^ CGM Clarity software (Dexcom, Inc., San Diego, CA, USA): the graph shows the glucose trend averaged over 14 days. No evidence of hypoglycemia in the early hours of the morning was found in the months following steroid replacement.

Nowadays, our patient is 15 years old, and her insulin requirement has doubled when compared with the daily need at the diagnosis of adrenal insufficiency. Currently, CMG data do not show any recurrence of hypoglycemia near dawn (Figure 2).

## 3. Discussion

The autoimmune diseases more frequently associated with type 1 diabetes are celiac disease and autoimmune thyroiditis [9]. Adrenal insufficiency and atrophic gastritis have been rarely observed; however, both deserve a timely diagnosis. Decades ago, latent Addison’s disease was described as follows: ‘A latent insufficiency always manifests in situations of stress, such as infection, overexertion, surgery, and trauma’. However, the patients are still capable of leading a normal life without maintenance therapy. They may feel healthy, and even the important symptom of fatigue may be absent or inconspicuous [21,22]. Adrenal insufficiency preceded by long-term autoantibody positivity could lead to a life-threatening event that sometimes represents the first clinical manifestation of Addison’s disease [10,11,12,13]. Thus, periodic screening of all autoimmune diseases is recommended for pediatric patients with type 1 diabetes [14,15].

Our patient’s clinical picture was compatible with autoimmune polyendocrine syndrome type 2 (APS 2), or Schmidt’s syndrome [16]. APS 2 is a complex polygenic disorder associated with the presence of specific class II HLA. It usually occurs in adulthood and is more frequent in females (male-to-female ratio, 1:3). Addison’s disease is the predominant and most stable component (anti-adrenal-gland antibodies in 80–90% of cases) associated with autoimmune thyroiditis and/or type 1 diabetes.

Other autoimmune disorders, even though less frequent, include autoimmune gastritis, pernicious anemia, myasthenia gravis, rheumatoid arthritis, immune thrombocytopenic purpura, Sjögren’s syndrome, gonadal failure, vitiligo, alopecia, and serositis [13]. Each disease can develop independently, even years or decades later; therefore, a lifelong screening is recommended to make a prompt diagnosis of potentially severe events related to this syndrome.

The clinical manifestations of primary adrenal insufficiency result from deficiency of all adrenocortical hormones (aldosterone, cortisol, androgens), the lack of which leads to the development of nonspecific symptoms (e.g., asthenia, appetite loss, nausea), which can be shared with other concurrent autoimmune conditions. For this reason, especially in its early stages, the diagnosis and treatment of this condition may be delayed. Hypoglycemia can be the presenting sign in children with adrenal insufficiency, and it can lead to the deterioration of glycemic control and the need for reduction of the total daily insulin dose in patients with type 1 diabetes. A specific sign of chronic, but not acute, primary adrenal insufficiency is hyperpigmentation, which predominantly affects areas of the skin subjected to pressure (elbows, knuckles, palmar creases, lips, buccal mucosa). A life-threatening adrenal crisis can be the first presentation of adrenal insufficiency. The clinical features include vomiting, abdominal pain, myalgia, joint pains, severe hypotension, and hypovolemic shock with electrolyte imbalance (hyperkalemia, hyponatremia). The acute presentation can be precipitated by physiological stress, such as surgery, trauma, or an intercurrent infection [17]. Clinicians should have a high index of suspicion for this condition, not only in patients already diagnosed with Addison’s disease, but also in those with a family history of any autoimmune endocrine gland failure or with one autoimmune endocrine disease who develop nonspecific or serious illness [19].

**Figure 3:**
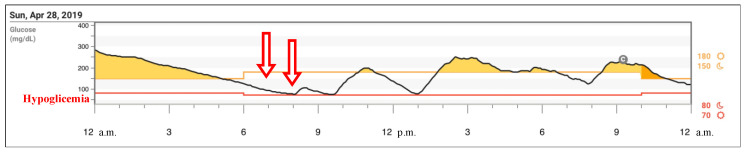
Glucose data reproduced from Dexcom^®^ CGM Clarity software (Dexcom, Inc., San Diego, CA, USA): the graph shows an example of daily glycemic excursions and evidence of hypoglycemia in the early hours of the morning.

As highlighted in this study, a periodic and careful evaluation of our patient’s CGM data allowed us to suspect the association of Addison’s disease, although latent and paucisymptomatic, specifically for the tendency toward low glucose levels in the late hours of the night, despite the administration of carbohydrates (Figure 3).

These observations, along with the extremely reduced daily insulin requirement, not appropriate for age and pubertal stage, and the presence of another autoimmune endocrinopathy, were interpreted as red flags, suggesting further investigation of adrenal function.

## 4. Conclusions

CGM is nowadays a widespread and pivotal tool offering the opportunity not only of a better management and quality of life for type 1 diabetes patients but also of early suspicion of the condition of latent adrenal insufficiency.

As suggested by this report, CMG is essential not only to limit potentially recurrent life-threatening hypoglycemic episodes, especially in patients susceptible to other autoimmune disorders, but also to suspect other autoimmune underlying conditions, such as latent or paucisymptomatic Addison’s disease, since their clinical manifestation may be sudden and leads to adrenal crisis during stressful events.

## Data Availability

Restrictions apply to the availability of these data. Data was obtained from online database (Dexcom Clarity)and are available from: https://www.dexcom.com/about-dexcom (accessed on 16 May 2021) with the permission of Dexcom Clarity.
